# Exploring faradaic and non-faradaic electrochemical impedance spectroscopy approaches in Parkinson's disease diagnosis

**DOI:** 10.1016/j.heliyon.2024.e27433

**Published:** 2024-03-05

**Authors:** Hussaini Adam, Subash C.B. Gopinath, Tijjani Adam, Makram A. Fakhri, Evan T. Salim, Sreeramanan Subramaniam

**Affiliations:** a Institute of Nano Electronic Engineering, Universiti Malaysia Perlis (UniMAP), 01000 Kangar, Perlis, Malaysia; b Faculty of Chemical Engineering & Technology, Universiti Malaysia Perlis (UniMAP), 02600 Arau, Perlis, Malaysia; c Micro System Technology, Centre of Excellence (CoE), Universiti Malaysia Perlis (UniMAP), Pauh Campus, 02600 Arau, Perlis, Malaysia; d Centre for Chemical Biology, Universiti Sains Malaysia, Bayan Lepas, 11900, Penang, Malaysia; e Department of Computer Science and Engineering, Faculty of Science and Information Technology, Daffodil International University, Daffodil Smart City, Birulia, Savar, Dhaka 1216, Bangladesh; f Faculty of Electronic Engineering & Technology, Universiti Malaysia Perlis, 02600, Arau, Perlis, Malaysia; g Laser and Optoelectronics Eng. Department, University of Technology-Iraq, Baghdad, 10066, Iraq; h Applied Science Department, University of Technology-Iraq, Baghdad, 10066, Iraq; i School of Biological Sciences, Universiti Sains Malaysia, Georgetown, 11800, Penang, Malaysia

**Keywords:** Parkinson's disease, Biomarker, EIS, Faradaic EIS, Non-faradaic EIS

## Abstract

Parkinson's disease is a neurodegenerative condition defined by the progressive death of dopaminergic neurons in the brain. The diagnosis of Parkinson's disease often uses time-consuming clinical evaluations and subjective assessments. Electrochemical Impedance Spectroscopy (EIS) is a useful technique for electroanalytical devices due to its label-free performance, in-situ measurements, and low cost. The development of reliable diagnostic tools for Parkinson's disease can be significantly enhanced by exploring novel techniques like faradaic and non-faradaic EIS detection methods. These techniques have the ability to identify specific biomarkers or changes in electrochemical properties linked to Parkinson's disease, allowing for an early and accurate diagnosis. Faradaic EIS detection methods utilize redox processes on the electrode surface, while non-faradaic EIS methods rely on charge transfer or capacitive properties. EIS can identify biomarkers or changes in electrical properties as indicators of Parkinson's disease by measuring impedance at different frequencies. By combining both faradaic and non-faradaic EIS approaches, it may be possible to obtain a comprehensive understanding of the electrochemical changes occurring in Parkinson's disease patients. This may lead to the development of more effective diagnostic techniques and potentially opening up new avenues for personalized treatment strategies. This review explores the current research on faradaic and non-faradaic EIS approaches for diagnosing Parkinson's disease using electrochemical impedance spectroscopy.

## 1 Introduction

**Fig. 1:**
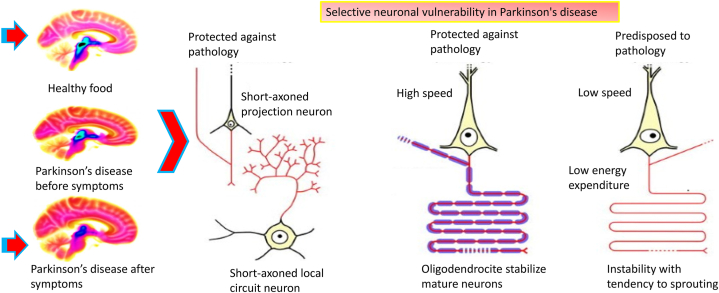
**Selective neuronal vulnerability in Parkinson's disease:** Parkinson's disease is characterized by selective neuronal susceptibility, where certain neurons are more susceptible to degeneration. Dopaminergic neurons in the substantia nigra are particularly affected, while others remain largely unaffected. The fundamental causes of this selective susceptibility remain unknown and are being investigated in Parkinson's disease research.

**Fig. 2:**
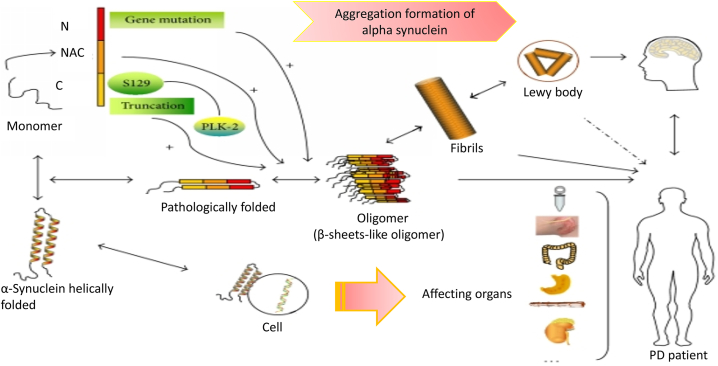
**Aggregation formation of alpha synuclein in Parkinson's disease:** Alpha synuclein aggregation into Lewy bodies is a clinical feature of Parkinson's disease. It is commonly acknowledged that β-sheet-rich αS oligomers contribute to the neurodegeneration seen in Parkinson's disease. Indeed, familial PD genetic abnormalities are found primarily in the S encoding gene, and such mutations increase the formation of poisonous αS oligomers in neurons. The neuropathological trait of alpha-synuclein aggregation in numerous cell types across the brain is shared by dementia with Lewy bodies, Parkinson's disease, and multisystem atrophy.

Parkinson's disease is a neurodegenerative disorder affecting the elderly, causing selective vulnerability of neurons, leading to gradual deterioration and impaired functioning of specific neuronal cells (Fig. 1) [1]. Parkinson's disease is a condition characterized by the selective vulnerability of dopaminergic neurons in the substantia nigra, which regulate movement and reward [2]. However, other types of neurons, such as short-axoned projection neurons and short-axoned local circuit neurons, are also affected. These neurons are responsible for transmitting signals over long distances and are particularly vulnerable in Parkinson's disease, leading to disruptions in communication and contributing to motor symptoms. Dysfunction of these neurons can result in cognitive impairments and non-motor symptoms. These neurons are supported by byoligodendrocytes, which are crucial in stabilizing mature neurons and protecting them from pathology. They provide myelin sheaths, promote efficient electrical signal transmission, and offer metabolic support, ensuring structural integrity. Researchers have identified specific patterns of neuronal death, however the underlying processes underlying this susceptibility remain unknown. Alpha synuclein oligomers are crucial in Parkinson's disease aetiology, as they impair cellular functions and lead to the onset of the condition. Located in presynaptic terminals, their aggregation is essential (Fig. 2) [3]. Alpha synuclein aggregation occurs when alpha synuclein proteins create abnormal clumps or aggregates in different organs, such as the heart and intestines. These aggregates can disturb normal cellular function and lead to the development of diseases including Parkinson's disease. The development of beta-sheet-like oligomers of alpha synuclein can cause the proteins to aggregate [4]. These aggregated proteins can disrupt the correct function of cells in the heart and stomach, potentially resulting in cardiac and gastrointestinal diseases. Furthermore, the helically folded monomeric form of alpha synuclein can aggregate and contribute to the degenerative processes seen in the heart and gut [5]. Parkinson's disease, a brain disorder characterized by the gradual loss of dopaminergic neurons, can lead to significant movement impairments that significantly affect daily activities [6]. These impairments include quivering or tremoring at rest, feeling sluggish or slow in movement, and having inflexible musculature that makes some tasks difficult to complete. Others include instability or difficulty keeping a good posture [7]. Parkinson's disease diagnosis using faradaic and non-faradaic EIS techniques is one approach that has showed potential [8]. This review explores the use of both faradaic and non-faradaic EIS approaches for diagnosing Parkinson's disease, aiming to improve the accuracy and sensitivity of detecting biomarkers. This may lead to earlier diagnosis and improved patient outcomes. Faradaic and non-faradaic EIS techniques are used to diagnose Parkinson's disease by identifying specific biomarkers or variations in compounds in biological samples [9]. Faradaic EIS technique involve charge transfer from an electrode to a redox active species, allowing the detection and identification of biomarkers like dopamine and its metabolites [10]. Non-faradaic EIS method rely on surface interactions between electrodes and target biomolecules to identify and measure their presence [11]. Parkinson's disease can be diagnosed using both faradaic and non-faradaic EIS approaches [12]. Faradaic EIS method offer a more specialized and accurate approach by directly measuring specific biomarkers linked to the condition [13]. This allows for early detection of individuals at risk for developing the disease, which is crucial for neuroprotective tactics and therapeutic interventions. Combining faradaic and non-faradaic EIS approaches can enhance the sensitivity of Parkinson's disease detection by utilizing electrical impedance changes related to the disease's presence and progression [14]. Non-faradaic EIS in label-free biosensors offers a cost-effective, simpler method for diagnosing Parkinson's disease, eliminating the need for labels or additional chemicals [15]. Furthermore, faradaic and non-faradaic EIS approaches can be used to detect Parkinson's disease by analyzing electrical impedance spectroscopy of biological samples [16]. These techniques can identify specific biomarkers associated with the disease, allowing for early and accurate diagnosis. They also help monitor disease progression and evaluate treatment efficacy, offering a non-invasive and cost-effective alternative to traditional diagnostic methods like imaging or genetic testing.

## 2 Electrical impedance spectroscopy

**Fig. 3:**
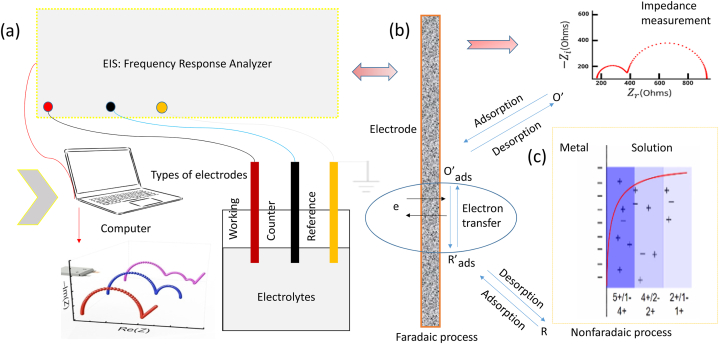
**Faradaic vs nonfaradaic EIS:**: The Frequency Response Analyzer is a crucial instrument in electrochemical impedance spectroscopy, analyzing a system's frequency response using a sine wave. It is widely used in monolithically integrated electrochemical sensors and semiconductor-fabricated instrumentation circuits. EIS, or Electrochemical Impedance Spectroscopy, is essential for ion transport and reactions at electrode/electrolyte interfaces. There are two methods: faradaic and non-faradaic EIS. Non-faradaic EIS investigates system responses without electrochemical processes, while faradaic EIS focuses on charge transfer mechanisms and redox reactions in electrochemical cells.

Electrical Impedance Spectroscopy is a powerful technique used in various fields such as biomedical engineering, material science, and chemical engineering [17]. EIS studies the impedance response across a broad frequency range by passing a modest amplitude AC signal through a system (Fig. 3a) [18]. This enables the study of different characteristics, including resistance, capacitance, and inductance, which may be used to evaluate the behaviour and performance of the system under consideration. EIS uses frequency response analyzer for enabling precise measurement and analysis of an electrochemical system's impedance across various frequencies. It helps understand electrochemical processes like corrosion, battery performance, and reaction kinetics. By applying AC voltage at different frequencies and measuring the resulting electrical current, it determines impedance. EIS measurements can be conducted using two methods: Faradaic EIS and non-faradaic EIS, with faradaic EIS utilizing a redox-active species for electrochemical reactions at contact (Fig. 3b) [19]. This reaction contributes to the overall impedance of the system and provides information about the processes occurring at the interface. Non-faradaic EIS is based on changes in interfacial capacitance caused by biomolecules flowing through a bio-functionalized electrode attached to a target molecule (Fig. 3c) [20]. For the characterization and investigation of electrochemical systems, notably in biosensors, non-faradaic EIS is a useful technique. During measurement, it involves charge transfer over electrode interfaces and tracks changes in capacitance. EIS is very helpful in biosensors since it offers in-situ measurements and nondestructive sensing. It can precisely measure changes at interfaces during recognition events, evaluating the electrodes' structural and functional characteristics. Due to its capability to deliver nondestructive sensing and in-situ measurements, EIS is useful in biosensors.

## 3 Experimental methods in electrochemical impedance spectroscopy

Electrochemical impedance spectroscopy is an experimental technique that assesses the transfer function of an electrochemical system by using a sinusoidal input signal that has been changed by voltage [21]. Electrochemical impedance spectroscopy measures the current response of a low-amplitude sinusoidal input signal, aiding in characterizing electrochemical systems like batteries, fuel cells, corrosion, and biological systems [22]. Electrochemical impedance spectroscopy is a critical tool in many research fields, including photovoltaic cells, bioelectric impedance analysis, tissue characterization, and meat quality control [22]. Electrochemical impedance spectroscopy is utilized for investigating transport, charge transfer, interface phenomena, conductivity, and diffusion in materials, photovoltaic cells, bioelectric impedance analysis, tissue characterization, and meat quality control [23]. Understanding the electrical properties of materials and systems, especially in electrochemistry, requires the use of experimental electrochemical impedance spectroscopy methods. With the use of these techniques, measuring electrode or electrochemical system impedance at different frequencies can enhance electrochemical processes at the electrode-electrolyte interface. These investigations are carried out using a variety of experimental procedures and techniques.

## 4 Interpretation and analysis of impedance spectroscopy data

**Fig. 4:**
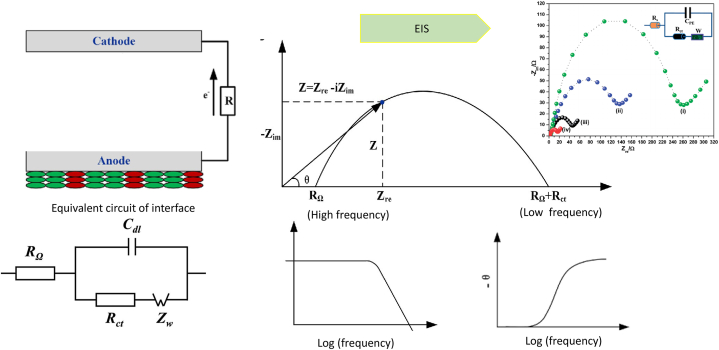
**The EIS interface circuit:** The EIS interface circuit is crucial for accurate impedance measurements in electrochemical impedance spectroscopy. It includes ohmic resistance, double-layer capacitance, and charge transfer resistance at high frequencies. At low frequencies, it includes Warburg impedance, finite diffusion of charged species, and bulk solution. At log frequency, it may include inductive or capacitive behavior.

Impedance spectroscopy is a powerful tool for studying the electrical properties of materials and their interactions [24]. Impedance spectroscopy is used to study frequency-dependent electrochemical behavior in various applications like dielectric materials, fuel cells, and batteries. However, selecting the right equivalent circuit and formalism for data analysis can be challenging. The choice of circuit determines the level of detail and accuracy in representing the system. The EIS interface circuit is crucial for accurate impedance measurements in electrochemical impedance spectroscopy (Fig. 4) [25]. At low frequencies, ohmic resistance, double-layer capacitance, and charge transfer resistance at high frequencies, Warburg impedance at low frequencies, finite diffusion of charged species within the electrode, and inductive or capacitive behaviour at log frequency all contribute to the impedance of an interface.

## 5 Detection of Parkinson's disease using electrochemical impedance spectroscopy techniques

**Fig. 5:**
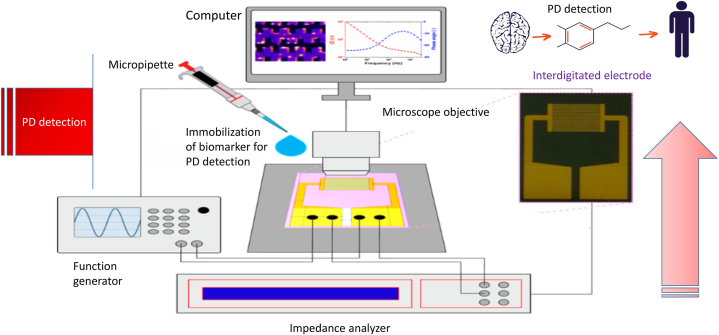
**EIS for Parkinson's disease measuring impedance with interdigitated electrodes:** Interdigitated electrodes are an EIS approach for Parkinson's disease, enhancing impedance measurement sensitivity by detecting changes in electrical properties and providing a higher surface area for molecular interaction. This leads to better chemical identification and adsorption efficiency.

**Fig. 6:**
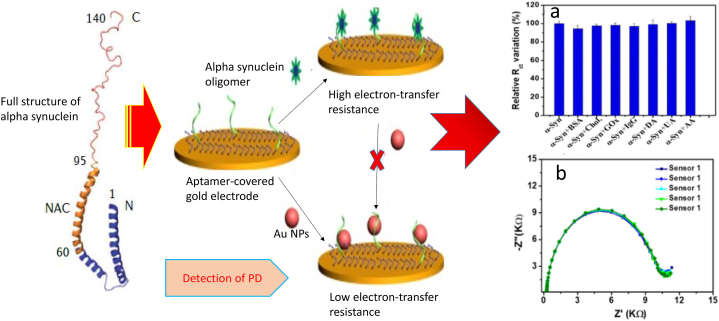
**EIS-based aptasensor for Parkinson's disease detection:** Recent interest in electrochemical biosensors for Parkinson's disease detection has led to the development of aptasensors, a promising option for detecting the disease. These biosensors use aptamers, single-stranded DNA or RNA molecules with high affinity and selectivity for binding to target molecules. They offer cost-effectiveness, high affinity, and specificity, enabling reliable detection of target biomarkers for Parkinson's disease.

Table 1 depicts electrochemical biosensors for Parkinson's disease detection that use different approaches to detect and evaluate biomarkers. These biosensors have great sensitivity, are compatible with miniaturisation methods, use little power, and are not affected by sample turbidity or colour. They precisely measure biomarkers such as dopamine levels, and the combination of nanotechnology with electrochemical biosensors enhances disease diagnosis and therapeutic management. Electrochemical biosensors have tremendous potential for detecting and monitoring Parkinson's disease. Impedance measurement with interdigitated electrodes is one approach of using EIS for Parkinson's disease (Fig. 5 [26]. Interdigitated electrodes, which are made up of parallel, closely spaced fingers, improve measurement sensitivity by detecting changes in the electrical characteristics of the electrode contact. They increase the surface area available for molecule interaction, improving chemical identification and adsorption effectiveness. The use of electrical impedance spectroscopy and interdigitated electrodes for biomarker PD detection has the potential to revolutionize early diagnosis and management of Parkinson's disease. This non-invasive technique can detect changes in specific biomarker properties, providing insights into PD progression and enabling early intervention. By monitoring changes in impedance, real-time information on biomarker levels can be provided, enabling timely adjustments in treatment plans and personalized care. Interdigitated electrodes enhance sensitivity and specificity by maximizing surface area for biomarker interaction. Frequency analyzers can also be used to analyze biomarker electrical behavior. In detecting alpha synuclein oligomers, an EIS-based aptasensor showed good selectivity and sensitivity (Fig. 6) [27]. EIS is a technique that uses an aptamer-covered gold electrode and electrical impedance spectroscopy to detect and quantify alpha synuclein oligomers, providing precise measurements of levels, potentially offering insights into neurodegenerative diseases like Parkinson's. The sensitivity of EIS response system is demonstrated in Fig. 6. An electrochemical nanobiosensor has been developed to aid in early detection and monitoring of Parkinson's disease and other synucleinopathies by utilizing biological recognition components in analytical equipment [28]. EIS provides electrochemical investigation of the electrical properties of the electrode surface. EIS in biosensor technology detects the kinetics of molecules such as DNA, receptors, antibodies, antigens, and proteins, as well as changes in biosensors on electrode surfaces [29]. This advantage makes studying biological binding kinetics easier. The impedance spectra obtained for molecule binding kinetics are used to carry out label-free detection. In enzyme-based biosensors, an enzyme must transform one molecule into another in order to create electroactive molecules or electrons [30]. Other molecules with the same oxidation reduction potential as the analyte molecule might disrupt the electroactive signal. This problem can be solved using electrochemical impedance spectroscopy, which provides non-electroactive molecule sensing [31]. As a result, the use of EIS in the diagnosis of Parkinson's disease will be examined. EIS-based biosensor systems might be utilized to detect biomarkers for Parkinson's disease, perhaps leading to the creation of feasible tests for diagnosis. Aptasensors, for example, have been designed to precisely detect alpha-synuclein oligomers in Parkinson's disease using electrochemical impedance spectroscopy and colorimetry [32]. The detection limits were found to be 10 nM, 8 pM, and 1 pM, respectively. An electrochemical biosensor based on a molecular fingerprint polymer has been created to detect alpha synuclein in Parkinson's disease [33]. The proposed sensor demonstrated exceptional analytical performance in the detection of alpha-synuclein, with a linear range of 1 fM to 10 pM, based on electrochemical impedance spectroscopy studies. Immunosensors have also been used to identify biomarkers for Parkinson's disease in the human body [34]. They were able to achieve a detection limit of 5.1 10-6 mol L-1. When the electrode was functionalized with specific anti-PARK7/DJ-1 antibodies, electrochemical impedance spectroscopy was effectively employed to detect Parkinson's disease protein 7 (PARK7/DJ-1). Researchers created an electrochemical immunosensor based on self-assembled monolayer modified electrodes for label-free detection of alpha synuclein [35]. Current and charge transfer resistance for the redox process were recorded when alpha synuclein was connected to the immunosensor probe surface. The immunosensor demonstrated excellent repeatability, anti-interference capabilities, and alpha-synuclein detection recovery in diluted human blood samples. The levels of dopamine in Parkinson's disease patients' cell solutions was investigated using impedance analysis [36]. The results show that the impedance measured during cell immobilisation is important because it shows the electrical response of the cell medium when a frequency is applied. Additionally, immunosensory testing using EIS has been used to identify biomarkers for Parkinson's disease [37]. The sensor and immunological sensor were used to examine the levels of Parkinson's disease biomarkers in the human body, and they showed to be a viable alternative method of diagnosing the disease. EIS experiments have also been done at frequencies ranging from 0.1 Hz to 100 kHz and amplitudes ranging from 5 mV to 10 mV to discover biomarkers for Parkinson's disease [38]. Furthermore, foldable platinum electrochemical and immunosensory electrodes based on EIS detection for the detection of a Parkinson's disease biomarker were created [34]. Electrochemical impedance spectroscopy was successfully used to identify Parkinson's disease protein 7 after electrodes were functionalized with particular anti-PARK7/DJ-1 antibodies [39]. Electrochemical impedance spectroscopy used alpha synuclein as a biomarker to detect Parkinson's disease, with detection limits of 3.62 and 1.13 ng/mL [40]. The detection limit for Parkinson's disease using electrochemical impedance spectroscopy with the biomarker alpha synuclein was 3.3 aM [41]. Furthermore, using the voltammetry method, Parkinson's disease was detected in both faradaic and non-faradaic EIS [42]. The sensitivity was 0.3 nA pM1 and the detection limit was 168 aM. The method was able to distinguish between matched and mismatched miR sequences, indicating a potential future in vitro application. Also, Parkinson's disease was detected using EIS. The performance of the electrodes was greatly enhanced by electroplating platinum black and reduced graphene oxide nanocomposites onto the electrode surface. This resulted in increased sensitivity, selectivity, and linearity as well as decreased impedance. Graphitic carbon nitride sensor was used for a direct and sensitive electrochemical assessment of pramipexole for Parkinson's disease [43]. For the selected concentration range of 0.5–30 M, a lower detection limit of 0.012 M was obtained, and with a linearity range of 0.05–500 M. The analysis's high recovery results demonstrate the electrode's effective performance. Electrochemical impedance spectroscopy is a valuable noninvasive tool for diagnosing and monitoring Parkinson's disease, detecting early stages and potentially tracking disease progression [44]. It analyzes the electrical properties of biological tissues, providing insights into pathological changes associated with Parkinson's disease. This method is cost-effective and patient-friendly, as it doesn't require expensive equipment or invasive procedures. It can track disease progression over time, providing clinicians with valuable information about treatment effectiveness. This integration of impedance spectroscopy into Parkinson's disease diagnosis and monitoring offers numerous advantages, including early detection, effective disease management strategies, personalized treatment approaches, and reduced burden of invasive procedures and costly equipment. However, it faces limitations such as the need for specialized equipment, potential variability in results due to electrode placement and patient characteristics, and lack of standardized data analysis protocols. EIS may also face challenge in differentiating Parkinson's disease from other neurological disorders with similar symptoms, potentially leading to misdiagnosis. Further research is needed to address these limitations and develop standardized protocols for EIS in Parkinson's disease diagnosis.

**Table 1:** Electrochemical biosensors for the detection of Parkinson's disease.

Biosensor Type	Chemical Process	Experiment	Fluid	Biomarker	LOD	Ref
Composition of poly(anilineboronic acid)/carbon nanotubes	Oxidation of dopamine.	In vitro experimentation	blood	Dopamine	–	[34]
Reduced graphene oxide composites with copper sulphide.	compound reaction based on CuS/RGO	In vitro experimentation	–	H_2_O_2_	–	[35]
Gold electrode nanocomposites with cysteamine and graphene.	Carboxylic acid forms covalent bond.	In vitro experimentation	Serum	Alpha synuclein	1.2-pM	[36]
Nanocomposites made from graphene, gold, and Fe3O4	Catalyst for Pt RGO/AuFe3O4-GCE reaction	In vitro experimentation	Tumour and normal cells	H_2_O_2_	0.1-μM	[37]
Nonenzymatic organic field-effect transistor biosensor	Oxidation of dopamine	In vitro experimentation	ISF	l-Dopa	10-pM	[38]
Hollow MN orthogonal electrochemical/biocatalytic	Oxidation of dopamine	In vitro/In vivo experimentation	ISF	l-Dopa	–	[39]
Platinum nanocomposite	oxidation of glutamate	In vitro experimentation	Spinal cord sample	Glutamate	0.2–0.5 μM	[40]
LDHs and graphene-based nanomaterials	Oxidation of dopamine	In vitro experimentation	Biological cells	Dopamine	2.0 nM	[41]
5-phenyl pentanamide	A single-step amide reaction.	In vitro experimentation	Blood	H_2_O_2_	0.02 μM	[42]
Nanobiosensor integrated using solid-phase microextraction method	Oxidation of dopamine	In vitro experimentation	Cytoplasm of a living cell	Dopamine	10pM	[43]
Wearable electrochemical platform for electrochemical reactions	Oxidation of l-Dopa	In vitro/In vivo experimentation	Sweat/Blood	l-Dopa	–	[44]
Biosensor combines N-doped carbon nanorods with Au nanoparticles	Oxidation of dopamine	In vitro experimentation	Serum	Dopamine	–	[45]
CPBMCPE biosensor with modified carbon paste electrode	Dopamine and uric acid undergo voltammetric oxidation	In vitro experimentation	Urine	Uric Acid/Dopamine	38–42 μM	[46]

## 6 Other biomarkers for Parkinson's disease diagnosis

Other biomarkers, such as alpha-synuclein, DJ-1 protein, nucleotide ligase chain, microRNAs, mitochondrial DNA, and metabolites such as uric acid and dopamine, may be used to enhance diagnostic techniques for Parkinson's disease, which have shown potential in various studies [45]. These biomarkers have shown potential in various studies. They could provide additional information for early detection, monitoring disease progression, and assessing treatment response in Parkinson's disease patients. Additional biomarkers to consider include inflammatory markers like cytokines and chemokines, oxidative stress markers like glutathione peroxidase and superoxide dismutase, and neurodegeneration markers like tau protein and amyloid-beta [46]. Also, changes in gut microbiota composition and function, along with examining neuronal imaging techniques like positron emission tomography scans and diffusion tensor imaging, could aid in developing more comprehensive diagnostic tools [47].

## 7 Challenges in electrochemical impedance spectroscopy

Electrochemical impedance spectroscopy is a valuable tool for researchers, however, it presents challenges in conducting experiments and analyzing data. One of the challenges is determining a suitable equivalent circuit to represent the electrochemical system under investigation. An equivalent circuit is a reduced model of a system that enables for electrical behaviour. Choosing the proper equivalent circuit can be difficult since it necessitates an in-depth understanding of the electrochemical processes and phenomena at work in the system. Another problem is determining the most suitable formalisms for data analysis. Formalisms are mathematical and analytical methodologies for extracting significant information from impedance spectroscopy data. Depending on the system and information needed, several formalisms may be required. Fitting experimental data to equivalent circuit models, for example, might give significant insights, although advanced computational approaches may be required for complex data sets. Because of the complexity of electrochemical processes and the unreliability of simple equivalent circuit models, interpreting impedance data may be difficult. To guarantee reliable data analysis and interpretation, researchers must employ proper approaches. This review suggests solutions for impedance analysis challenges, including refined equivalent circuit models, advanced data analysis formalisms, and simulation-based methods. These methods aim to improve the accuracy and reliability of impedance analysis, enabling researchers to gain a deeper understanding of the electrochemical system under investigation. Other solutions include advancements in measurement techniques, data processing, and machine learning algorithms. These methods can enhance the accuracy of impedance analysis and extract valuable insights from complex datasets. In conclusion, addressing impedance analysis challenges requires a combination of these solutions.

## 8 Future research directions in faradaic and non-faradaic EIS diagnosis methods

More study is needed to improve the sensitivity, selectivity, and reliability of faradaic and non-faradaic EIS Parkinson's disease tests for diagnosis. This might entail investigating new redox active compounds for faradaic EIS method as well as creating new non-faradaic EIS strategies for increased selectivity in biomarker detection. Integrating these approaches with cutting-edge technology such as microfluidic devices and biosensors might improve the accuracy and efficiency of Parkinson's disease diagnosis, allowing for real-time monitoring and personalized treatment options. The invention and optimisation of both faradaic and non-faradaic EIS have the potential to improve patient outcomes and diagnose Parkinson's disease early, therefore revolutionising clinical diagnostics. Electrochemical impedance spectroscopy (EIS) is a crucial diagnostic tool in fields like material science, electrochemistry, and biomedical engineering. Future research should focus on developing new electrode materials with improved conductivity and stability, exploring new measurement techniques for real-time EIS monitoring, and understanding the underlying electrochemical processes. The integration of machine learning algorithms and artificial intelligence techniques is also needed for improved data analysis and interpretation. Non-faradaic EIS methods, such as impedance-based biosensors and label-free detection techniques, can provide rapid and sensitive detection of biomarkers and analytes. These advancements will lead to more efficient and accurate EIS diagnosis methods, potentially enhancing applications in healthcare, environmental monitoring, and energy storage.

## 9 Conclusion

Advancements in both faradaic and non-faradaic approaches have the potential to significantly impact clinical diagnostics and patient outcomes for Parkinson's disease. Faradaic approaches, such as electrochemical sensing techniques, offer high sensitivity and specificity, enabling early detection and monitoring of disease progression with unprecedented precision. These advancements also have the potential to enhance the overall quality of life for individuals living with Parkinson's disease. Non-faradaic approaches, such as optical and acoustic methods, provide non-invasive and real-time assessment of physiological changes associated with Parkinson's disease, offering a more patient-friendly and continuous diagnostic experience. Earlier and more accurate diagnosis may lead to earlier interventions and personalized treatment plans, improving the management of symptoms and potentially slowing disease progression. Continuous monitoring of disease biomarkers through non-invasive methods may offer a more comprehensive understanding of the disease trajectory, leading to more tailored and effective interventions. These cutting-edge approaches have the potential to transform disease detection and monitoring, providing a more patient-friendly and continuous diagnostic experience. As these technologies advance, the potential to improve patient outcomes and redefine the standard of care for Parkinson's disease becomes more promising.

## CRediT authorship contribution statement

**Hussaini Adam:** Writing – review & editing, Writing – original draft, Validation, Investigation, Formal analysis, Data curation, Conceptualization. **Subash C.B. Gopinath:** Writing – review & editing, Visualization, Validation, Supervision, Software, Resources, Project administration, Funding acquisition. **Tijjani Adam:** Writing – review & editing, Validation, Software, Data curation. **Makram A. Fakhri:** Writing – review & editing, Visualization, Validation, Resources. **Evan T. Salim:** Writing – review & editing, Visualization, Validation. **Sreeramanan Subramaniam:** Writing – review & editing, Validation.

## Declaration of competing interest

The authors declare that they have no known competing financial interests or personal relationships that could have appeared to influence the work reported in this paper.
